# Resistance Exercise in People With Stage-3 Chronic Kidney Disease: Effects of Training Frequency (Weekly Volume) on Measures of Muscle Wasting and Function

**DOI:** 10.3389/fphys.2022.914508

**Published:** 2022-06-24

**Authors:** Louise J. Geneen, Jodie Kinsella, Tobia Zanotto, Patrick F. Naish, Thomas H. Mercer

**Affiliations:** ^1^ Centre for Health, Activity and Rehabilitation Research, School of Health Sciences, Queen Margaret University, Edinburgh, United Kingdom; ^2^ Department of Renal Medicine, University Hospital of North Staffordshire, Stoke-on-Trent, United Kingdom

**Keywords:** body composition, chronic kidney disease, muscle architecture, physical function, resistance training

## Abstract

**Background:** Resistance training (RT) is a proven anabolic intervention in people living with and without chronic kidney disease (CKD). To date, there is a dearth of knowledge regarding the dose-response relationship of RT in the non-dialysis dependent CKD population. Therefore, we aimed to explore the effects of RT frequency (weekly volume) on established measures of muscle wasting and function in CKD.

**Methods:** Twenty people with stage-3 CKD (CKD-3) were allocated to either a low frequency (one-session per week, RT1) or higher frequency (three-sessions per week, RT3) 12-week RT programme consisting of lower extremity strengthening exercises. The two RT programmes were not volume matched. Assessment outcomes before and after the intervention included measures of total and regional body composition, muscle size and architecture, strength, physical function, and uraemic symptoms.

**Results:** Significant improvements over time in muscle size and architecture, strength, physical function, and uraemic symptoms were observed for both RT1 and RT3. Compared to RT1, participants who performed RT3 showed greater increases in vastus lateralis (VL) anatomical cross-sectional area (30.8% vs. 13.2%, *p* < 0.001) and pennation angle (36.3% vs. 17.5%, *p* = 0.008) after 12 weeks. In either group, there were no significant changes over time in mid-VL fascicle length, nor in measures of total body composition and upper arm muscle strength.

**Conclusion:** Despite the group differences observed in the VL physiological adaptations, the strength and physical function responses, as well as the reductions of uraemic symptoms, were similar whether training once or thrice weekly. Therefore, performing RT just once per week may be an effective pre-habilitation strategy for people with CKD-3.

## 1 Introduction

Frailty is more prevalent in individuals living with chronic kidney disease (CKD) compared to the age-matched non-uraemic population (Chowdhury et al., 2017). Frail people with CKD are started on dialysis earlier than those who are not frail (Nixon et al., 2018) and the deterioration trajectory in terms of independence in performing activities of daily living is increased from dialysis initiation (Sy and Johansen, 2017; Nixon et al., 2018). This underscores the need to intervene early to attenuate the physical function decline, and thus delay these patients reaching the stage of dialysis.

In healthy people, resistance training (RT) is an established anabolic intervention with multiple long-term benefits (Borde et al., 2015), and using RT to maintain physical function has also been recommended for frail older adults (Fragala et al., 2019). With ageing, preserving muscle mass and physical function is also fundamental for delaying the onset of chronic diseases, which is often evidenced by reduced needs for medical intervention (Matchar et al., 2017).

Resistance training (RT) has also been shown to decrease muscle catabolism and counteract weight loss due to malnutrition in non-dialysis dependent (NDD) CKD patients (Cheema et al., 2014). While it has been recommended that RT should be initiated early during the pre-dialysis stages (Gollie et al., 2018) there are currently very few published reports of RT in NDD patients (Cheema et al., 2014; Watson et al., 2015; Clyne and Anding-Rost, 2021; Gadelha et al., 2021).

Systematic reviews of exercise interventions in CKD have underlined the lack of randomised controlled trials investigating RT (Heiwe and Jacobson, 2011). In addition, interpreting the findings of these individual studies is challenged by the fact they implemented different training regimens (e.g., session durations, exercise intensities, intervention lengths) and reported results by employing a range of different measures for similar outcomes (Heiwe and Jacobson, 2011; Clyne and Anding-Rost, 2021). Therefore, further research on the effects of RT frequency and intensity is warranted to shed more light on the greatest dose-response relevant to individuals with CKD, as well as to develop and prescribe the most effective interventions in the CKD population (Smart et al., 2013).

The aim of this study was to examine the effectiveness of RT as a preventive measure against the CKD-related muscle wasting in people with stage-3 CKD (CKD-3). We aimed to explore the effects of training frequency on whole and regional body composition, muscle size and architecture, measures of strength and physical function, and self-reported uraemic symptomatology. We hypothesised that the effects of exposure to the RT programme once per week (RT1) would be significantly different from exposure to the intervention three times per week (RT3).

## 2 Materials and Methods

### 2.1 Participants

Forty-five people living with CKD-3 were approached during a routine outpatient clinic, out of which 20 provided written informed consent and took part in the baseline assessments. Inclusion criteria were: age 18-years or older, confirmed diagnosis of CKD-3 (moderately reduced kidney function), glomerular filtration rate (GFR): 30–59. Participants were independently mobile, fluent in written and spoken English, and did not have prior experience of RT. Exclusion criteria were: diagnosis of unstable angina, amputations, and any known skin conditions or allergies (potentially affected by the ultrasound conductive jelly). The study conformed to the ethical principles for medical research involving human participants, as outlined by the world medical association declaration of Helsinki and all procedures were approved by the local National Health Service ethics board and Queen Margaret University ethics committee. Participants provided written informed consent prior to their participation.

### 2.2 Study Design and Setting

The baseline assessments (t_0_) took place at the Renal Rehabilitation gymnasium at the University Hospital of North Staffordshire. All assessments were made by a single investigator and included measurement of 1) total body composition; 2) regional body composition; 3) muscle architecture; 4) neuromuscular performance, including peak isometric strength; 5) physical function; and 6) uraemic symptoms. Following the baseline assessments, participants were randomly assigned using a computerised block randomisation programme to either a low frequency (one session/week, RT1) or higher frequency (three sessions/week, RT3) RT group. Participants in both groups performed lower extremity strengthening exercises for 12 weeks and returned to the study center for the post-intervention assessments (t_12_).

### 2.3 Intervention

Prior to the intervention, an initial estimate of participants’ one-repetition maximum (1-RM) was performed using the following equation for untrained individuals: 1-RM = 1.554 x (7–10-RM weight in kg)—5.181 (Braith et al., 1993). From the initial estimated 1-RM, weight was progressively added (if needed) in small increments of 0.5–2.5 kg until participants reached their actual 1-RM. A minimum rest interval of 1-min was allowed during successive lifting attempts. This 1-RM testing procedure lasted approximately 30 min and was performed for all exercises involving weight machines (described below).

Each training session was supervised and consisted of a 5-min aerobic warm-up on an unloaded cycle ergometer (Monark, Sweden) followed by lower extremity exercises using weight machines (leg press, knee extension, hamstring curl and calf raise) and bodyweight exercises (squats and lunges). During each session, participants completed three sets of eight repetitions at 80% of the 1-RM. Short recovery periods (1 minute) were allowed between sets, with a longer recovery period (2 minutes) between each different exercise. The main part of the intervention was followed by a short cool-down (same as aerobic warm-up) and general stretching of the trained muscle groups. The total session duration time was 1 hour. When participants were able to surpass the prescribed dose of three sets of eight repetitions, the 1-RM was accordingly adjusted. Knee extension and leg press peak force were used as a guide for progression.

### 2.4 Outcome Measures

#### 2.4.1 Total Body Composition

Body mass index (BMI) was calculated with the canonical formula [weight (kg)/height (metres)^2^]. Bioelectrical impedance analysis was used to measure fat/fat-free mass and total body water (Tanita MC-780-MA, Tanita Europe, Amsterdam). Biochemistry values were collected as part of the usual care of each participant by a trained nurse. The estimated GFR (ml/min/1.73 m^2^) was calculated with the following formula: GFR = 186 x (serum creatinine/88.4)^−1.154^ x (age)^−0.203^ x (0.742 if female) x (1.210 if black) (Levey et al., 1999).

#### 2.4.2 Regional Body Composition and Muscle Size/Architecture

Ultrasound measurements of regional body composition were taken with participants lying in a supine position for a minimum of 20-min to allow for any fluid shifts prior to assessment. Participants were instructed to relax the muscles and to keep the knee extension angle at zero degrees of flexion. A 50 mm, 7.7 MHz linear array probe (Sonosite^®^ 180 plus, Sonosite Inc., United States) was utilised to measure the vastus lateralis (VL) muscle thickness (depth), VL anatomical cross-sectional area (VL ACSA), VL pennation angle, VL subcutaneous fat thickness (depth), triceps brachii muscle thickness (depth), and triceps brachii subcutaneous fat thickness (depth). Ultrasound measures of the VL (muscle/fat thickness and pennation angle) were taken at half of the femur length and mid-VL width. Images were obtained by keeping the probe along the mid-sagittal line of the VL and perpendicular to the dermal surface. Fascicle length was calculated with the equation: VL *M*th/sin *θ*. Individual frame capture software (Adobe Premier v5.1, Adobe Systems) was used to acquire the VL ACSA images, which were collected in the axial plane and recorded onto SVHS videotape. In addition, measures of upper arm subcutaneous fat/muscle thickness (and handgrip strength) were also taken before and after the intervention, as a form of control data, to check whether any nutritional and/or pathophysiological adaptations occurred at a body-site that was not directly involved in the RT intervention. All measurements were performed by a single assessor highly experienced in ultrasonography. The intra-evaluator test-retest reliability and the minimal detectable changes of the ultrasound measurements have been fully described elsewhere (Geneen et al., 2022).

#### 2.4.3 Neuromuscular Performance (Strength)

Handgrip strength was assessed by means of a handheld dynamometer (Lafayette Instrument, Lafayette, IN). Isometric leg press peak force (LPPF) and isometric knee extension peak force (KEPF) were measured at 45°, as fully described by Gleeson et al. (2002). The best of three attempts on the dominant side was taken for analysis purposes.

#### 2.4.4 Physical Function and Physical Activity

Sit-to-stand 60 (STS60) and sit-to-stand 5 (STS5) tests were performed from a standard chair (height = 0.42 m) without arm rests. The North Staffordshire Royal Infirmary (NSRI) walk test consisted of performing a 50-m walk, a stair climb of 22 steps (with step height of 15cm and 3.3-m elevation), the same stair descent, and walk back to the starting point (Mercer et al., 1998). Participants were also asked about their physical activity levels during the last week (Physical Activity Recall) (Blair et al., 1985).

#### 2.4.5 Uraemic Symptoms

The Leicester Uraemic Symptom Score (LUSS) was used to evaluate uraemic symptomatology affecting the health-related quality of life (Pugh-Clarke et al., 2006). The LUSS is a five-point scale that assesses symptom number (LUSS 1, maximum score = 11), frequency (LUSS 2, maximum score = 44) and intrusiveness (LUSS 3, maximum score = 44) of eleven CKD-related symptoms (loss of muscle strength/power, pain in joints/bones, muscle spasm/stiffness, excessive tiredness, sleep disturbance, poor concentration/mental alertness, restless legs, shortness of breath, impotence/lack of sex drive, loss of appetite, and itching). The total LUSS score is summative (total maximum score = 99).

### 2.5 Statistical Analysis

Statistical analyses were performed with SPSS (Version 27.0 for Windows, SPSS Inc., Chicago, IL). The Shapiro-Wilk test was used to check whether data were normally distributed. Standard statistical methods were used for the calculation of mean and standard deviations (SD). Group comparison at baseline (RT1 vs. RT3) was assessed by Student’s unpaired *t*-test for all measures. Mixed model repeated measures ANOVA was employed to explore time and group effect and interactions. The homogeneity of variance was checked through the Levene’s test. Statistical significance was set at *p* < 0.05. Results are reported as mean and SD unless otherwise stated.

## 3 Results

**FIGURE 1:**
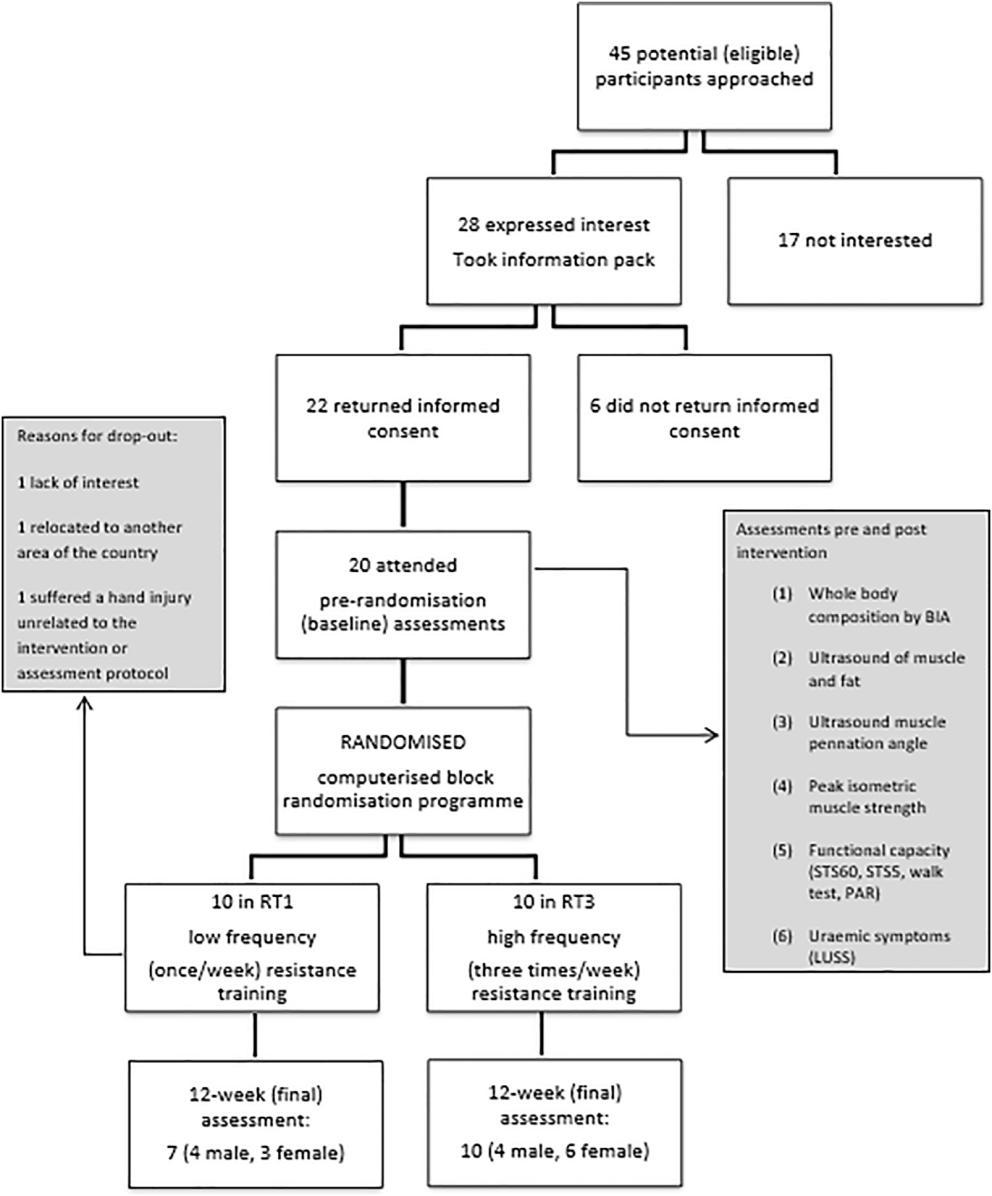
Participant flow chart. Legend: BIA: bioelectrical impedance analysis, STS5: sit-to-stand 5 test, STS60: sit-to-stand 60 test, PAR: Physical Activity Recall, LUSS: Leicester Uraemic Symptom Score.

The participant flow throughout the study is summarised in Figure 1. A total number of 17 individuals completed the 12-week intervention and were therefore included in the final analysis. At baseline, no significant differences (*p* > 0.05) between groups were observed in demographics and physical characteristics (Table 1). Main outcome measures from the intervention (Table 2) showed significant improvement over time (F_s_(1,15) = 5.209, p_s_<0.037) in muscle size, mid-VL fat depth, strength, function, and uraemic symptoms, with only group differences (group by time interaction) appearing in VL ACSA (F (1,15) = 25.059, *p* < 0.001) and pennation angle (F (1,15) = 9.438, *p* = 0.008) post-intervention. There were no significant changes over time or between groups in mid-VL fascicle length.

**TABLE 1:** Participant characteristics at baseline: results are presented as mean ± standard deviation.

	RT1	RT3
Sample size *n (M:F)*	7 (4:3)	10 (4:6)
Age (*years*)	52.9 ± 16.5 (*34–76*)	59.7 ± 9.9 (*44–73*)
(*range*)		
Ethnicity *n (%)*		
Caucasian	7 (100%)	9 (90%)
Asian	0 (0%)	1 (10%)
Weight (*kg*)	79.4 ± 9.1	75.8 ± 16.7
BMI (*kg/m* ^*2*^)	29.7 ± 4.7	27.3 ± 4.1
% fat	35.4 ± 11.7	33.25 ± 9.0
Fat mass (*kg*)	28.3 ± 10.4	25.0 ± 8.5
FFM (*kg*)	51.1 ± 9.3	50.8 ± 14.1
TBW (*L*)	37.4 ± 6.8	37.2 ± 10.3
CCr (*ml/min*)	49.1 ± 9.6	47.1 ± 6.5
SerumCr (*umol/L*)	135 ± 87	176 ± 45.5
Urea (*mmol/L*)	11.2 ± 6.3	15.0 ± 4.6
Albumin (*g/L*)	42.7 ± 2.2	40.5 ± 2.7
Hb (*g/dl*)	13.7 ± 1.4	13.0 ± 2.0
CO_2_ (*mmol/L*)	28 ± 2.3	26.4 ± 2.6
GFR (*ml/min*)	36.1 ± 12.9	36.6 ± 15.8

BMI, body mass index; FFM, fat free mass; TBW, total body water; CCr, creatinine clearance; Hb, haemoglobin; CO2, carbon dioxide in blood serum; GFR, glomerular filtration rate.

**TABLE 2:** Primary and secondary outcome measures pre- and post-intervention: results are presented as mean ± standard deviation.

		Pre-intervention (t_0_)			Post-intervention (t_12_)			Significance (*p*-value)	
		RT1 (*n* = 7)	RT3 (*n* = 10)	Total (*n* = 17)	RT1 (*n* = 7)	RT3 (*n* = 10)	Total (*n* = 17)	Time (Pre/post)	Group (RT1/RT3)
Ultrasound measures at mid-VL point	VL ACSA (*cm* ^*2*^)	19.2 ± 3.1	18.2 ± 6.9	18.6 ± 5.5	21.7 ± 3.2	23.9 ± 6.1	23.0 ± 5.1	<0.001	<0.001
	VL depth (*cm*)	2.5 ± 0.2	2.3 ± 0.4	2.4 ± 0.35	2.8 ± 0.3	2.5 ± 0.5	2.6 ± 0.4	<0.001	NS
	Total muscle depth (*cm*)	4.0 ± 0.3	3.9 ± 0.6	3.95 ± 0.5	4.5 ± 0.5	4.1 ± 0.8	4.3 ± 0.7	<0.001	NS
	Fat depth (*cm*)	1.3 ± 0.9	1.2 ± 0.7	1.3 ± 0.7	1.2 ± 0.8	1.1 ± 0.6	1.1 ± 0.7	0.008	NS
	VL pennation angle (*degrees*)	15.4 ± 2.3	16.8 ± 1.6	16.3 ± 2.0	18.1 ± 2.1	22.9 ± 3.2	20.9 ± 3.6	<0.001	0.008
	VL fascicle (fibre) length (*cm*)	3.4 ± 1.1	3.4 ± 1.5	3.4 ± 1.5	3.9 ± 1.0	4.5 ± 1.5	4.3 ± 1.4	NS	NS
Isometric muscle strength	Knee extension peak force 45 (*Newtons*)	266.1 ± 126.3	343.9 ± 114.1	311.9 ± 121.9	382.0 ± 138.2	420.4 ± 128.7	404.6 ± 129.8	<0.001	NS
	Leg press peak force (*Newtons*)	314.9 ± 161.1	217.2 ± 99.8	257.4 ± 133.4	413.8 ± 143.3	396.9 ± 160.0	403.9 ± 148.9	<0.001	NS
Physical Function and Activity	Sit-to-stand 5 (*secs*)	12.3 ± 2.0	12.4 ± 2.0	12.3 ± 4.2	8.0 ± 2.4	7.7 ± 2.4	7.9 ± 1.9	<0.001	NS
	Sit-to-stand 60 (*count*)	21.6 ± 5.9	25.3 ± 5.9	23.8 ± 6.7	34.1 ± 9.6	36.6 ± 9.6	35.6 ± 8.1	<0.001	NS
	NSRI walk test (*secs*)	90.8 ± 19.5	79.8 ± 15.3	84.4 ± 17.5	74.6 ± 20.6	70.0 ± 14.1	71.9 ± 16.6	<0.001	NS
	Physical Activity Recall (*kcal/kg/week*)	246.0 ± 11.2	255.1 ± 26.5	251.4 ± 21.5	253.8 ± 11.7	266.9 ± 10.5	261.5 ± 12.6	0.037	NS
Uraemic Symptoms	LUSS 1 (*number*)	6.7 ± 2.9	5.1 ± 2.9	5.8 ± 2.4	4.7 ± 3.6	4.5 ± 3.6	4.6 ± 2.7	0.003	NS
	LUSS 2 (*frequency*)	14.0 ± 9.0	13.1 ± 9.0	13.4 ± 7.8	7.7 ± 7.2	8.9 ± 7.2	8.4 ± 5.5	0.001	NS
	LUSS 3 (*intrusiveness*)	7.0 ± 5.9	6.5 ± 5.9	6.7 ± 4.8	3.1 ± 2.9	4.1 ± 2.9	3.7 ± 2.7	0.006	NS
	Total (*summative score*)	27.7 ± 16.3	24.7 ± 12.8	25.9 ± 13.9	15.6 ± 13.1	17.5 ± 8.0	16.7 ± 10.0	0.002	NS

VL, vastus lateralis; ACSA, anatomical cross-sectional area; NSRI, North Staffordshire Royal Infirmary Walk test; LUSS, Leicester Uraemic Symptom Score; NS, non-significant, *p* > 0.05.

### 3.1 Upper Body (Control Data)

Over the 12-week intervention, significant reductions in triceps brachii depth (F (1,15) = 5.758, *p* = 0.030), triceps fat depth (F (1,15) = 6.053, *p* = 0.026), and bicep fat depth (F (1,15) = 4.611, *p* = 0.049), without any significant interaction were found (Table 3). No significant changes over time or between groups were recorded for handgrip strength.

**TABLE 3:** Control data pre- and post-intervention: results are presented as mean ± standard deviation.

	RT1 t_0_	RT1 t_12_	RT3 t_0_	RT3 t_12_
Handgrip (*kg*)	25.9 ± 11.3	26.1 ± 10.7	28.5 ± 12.2	29.1 ± 13.5
Tricep brachii depth (*cm*)	4.3 ± 0.8	4.1 ± 0.8	3.9 ± 0.8	3.6 ± 0.9
Tricep fat depth (*cm*)	2.2 ± 1.2	2.2 ± 1.2	2.1 ± 0.8	1.9 ± 0.8
Bicep brachii depth (*cm*)	2.8 ± 0.8	2.8 ± 0.8	2.9 ± 0.6	2.9 ± 0.7
Bicep fat depth (*cm*)	1.9 ± 0.8	1.7 ± 0.8	1.7 ± 0.8	1.3 ± 0.6

Control data are ultrasound-derived measures of upper arm muscle thickness/depth and upper arm subcutaneous fat thickness/depth, and handgrip strength.

### 3.2 Total Body Composition

No statistically significant changes in measures of total body composition, as assessed by bioelectrical impedance analysis, were found (Table 4).

**TABLE 4:** Total body composition measurements pre- and post-intervention: results are presented as mean ± standard deviation.

	RT1 t_0_	RT1 t_12_	RT3 t_0_	RT3 t_12_
Weight (*kg*)	79.4 ± 9.1	81.1 ± 9.9	75.8 ± 16.7	78.1 ± 15.9
BMI (*kg/m* ^*2*^)	29.7 ± 4.7	30.3 ± 4.6	27.2 ± 4.1	28.1 ± 3.6
Fat (*%*)	35.4 ± 11.7	36.0 ± 10.9	33.3 ± 9.0	32.6 ± 10.6
Fat mass (*kg*)	28.3 ± 10.4	29.5 ± 9.6	25.0 ± 8.5	25.3 ± 9.4
FFM (*kg*)	51.1 ± 9.3	51.8 ± 9.0	50.8 ± 14.1	52.9 ± 14.5
FFM (*ratio*)	1.20	1.18	1.25	1.24
*Truncal/appendicular*	*27.9/23.2*	*28.0/23.7*	*28.2/22.6*	*29.3/23.6*
TBW (*L*)	37.4 ± 6.8	37.8 ± 6.7	37.2 ± 10.3	38.7 ± 10.6

BMI, body mass index; FFM, fat free mass; TBW, total body water.

Truncal/appendicular reflects the calculation of the FFM ratio in the row immediately above.

### 3.3 Regional Body Composition and Muscle Size/Architecture

**FIGURE 2:**
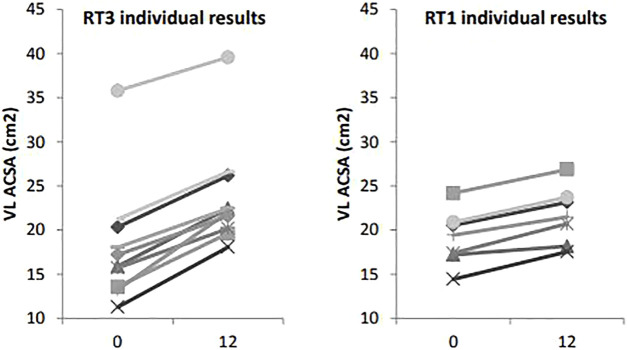
Vastus lateralis anatomical cross-sectional area measures at baseline (t0) and post-intervention (t12): individual changes in RT1 and RT3. Legend: VL ACSA: vastus lateralis cross-sectional area.

**FIGURE 3:**
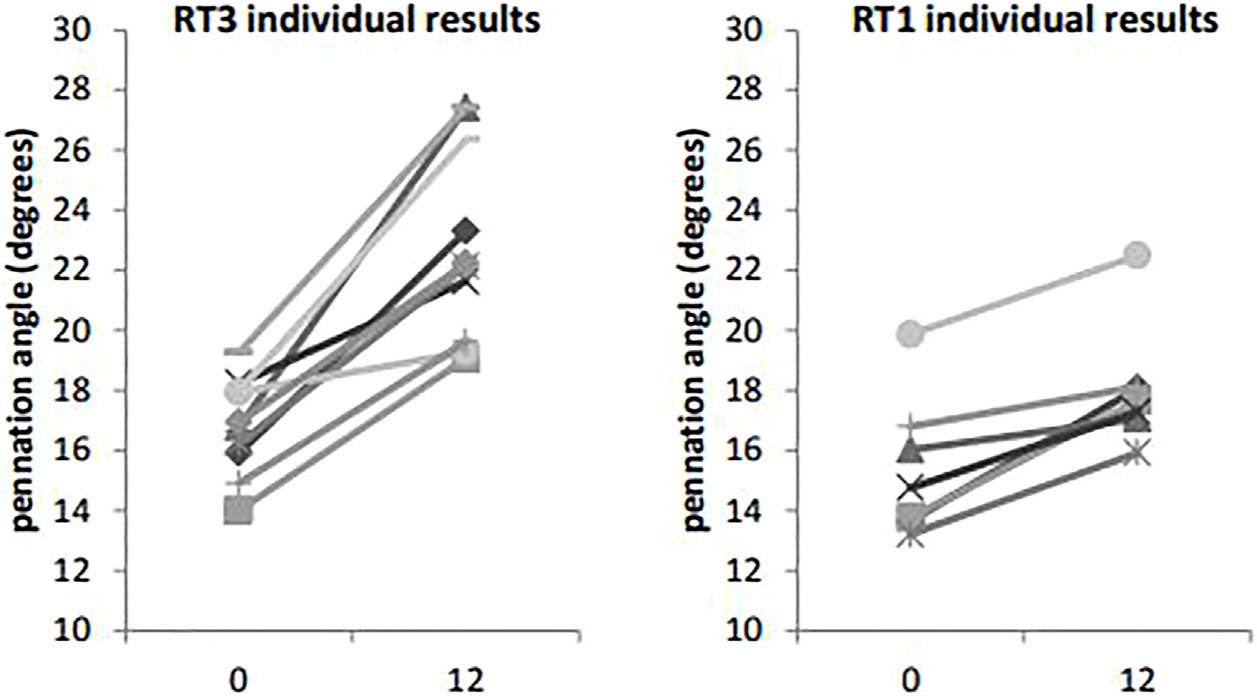
Vastus lateralis pennation angle at baseline (t0) and post-intervention (t12): individual changes in RT1 and RT3. Legend: VL: vastus lateralis.

VL ACSA exhibited significant changes over the 12-week period time, and between groups (Figure 2). Compared to RT1, RT3 increased VL ACSA by an additional 3.1 cm^2^ (Table 2). Such increase corresponds to a 30.8% increase in RT3 and 13.2% in RT1 over the intervention period. Figure 3 shows the individual changes in VL pennation angle in RT1 and RT3 after the intervention. Participants in RT3 increased their pennation angle from 16.8 ± 1.6° to 22.9 ± 3.2° over the 12 weeks, while participants in RT1 increased from 15.4 ± 2.3° to 18.1 ± 2.1°. This corresponded to a 36.3% increase in RT3, which was twice the increase found in RT1 (17.5%; Table 2). No statistically significant changes over time, nor group by time interactions, were recorded for fascicle length at the mid-VL point (Table 2).

### 3.4 Neuromuscular Performance (Strength)

Over the 12 weeks, both groups increased KEPF from 311.9 ± 121.9 N to 404.6 ± 129.8 N (F (1,15) = 25.182, *p* < 0.001), which corresponds to a 29.7% increase. In addition, both groups increased LPPF from 257.4 ± 133.4 N to 403.9 ± 148.9 N (F (1,15) = 51.007, *p* < 0.001), which corresponds to a 56.9% increase. However, no between-group differences were detected for either KEPF or LPPF (Table 2). While the LPPF scores appeared to be lower at baseline in the RT3 group compared to RT1 (217.2 ± 99.8 N vs. 314.9 ± 161.1 N), this difference was not statistically significant.

### 3.5 Physical Function and Physical Activity

The performance in STS5, STS60 and NSRI walk tests, as well as the Physical Activity Recall, improved over time in both groups (Table 2).

### 3.6 Uraemic Symptoms

Significant reductions in uraemic symptomatology (LUSS) were observed after the 12-week intervention without differences between the groups (Table 2).

## 4 Discussion

In the current study, we hypothesised that the effects of exposure to our RT programme once per week (i.e., RT1) would be significantly different from those participants who trained three times per week (i.e., RT3), as a dose-response relationship between exercise volume/frequency and strength has been described in young and healthy subjects, where higher training volume leads to increased muscle mass and strength (Schoenfeld et al., 2017). However, it has been proposed that individuals who are deconditioned as a result of chronic illnesses may have a training threshold. In other words, when individuals that are deconditioned meet a certain threshold-stimulus, after the initial exercise-related physiological adaptations, further increases in training frequency or volume may not translate into greater strength gains (Benton et al., 2011). For instance, many studies have concluded that performing RT just once per week results in similar benefits in terms of neuromuscular performance and physical function compared to training two or three times per week in non-uraemic older adults (Taaffe et al., 1999; DiFrancisco-Donoghue et al., 2007; Padilha et al., 2015; Turpela et al., 2017). The findings from this investigation are aligned with these studies, as there were no discernible differences between groups (RT1 vs. RT3) in measures of maximal muscle strength, physical function, and uraemic symptomatology. Nevertheless, statistically significant group differences were noted in some aspects of VL muscle size (ACSA) and architecture (pennation angle). Similarly, the apparent weight gain exhibited by participants after the intervention could be explained by the non-statistically significant increase in fat-free mass observed for RT3, which was three times higher compared to RT1 (Table 4). In this respect, performing RT three times per week may be more beneficial than training once a week for maintaining the critical muscle mass and reducing incidence of muscle cachexia in people living with CKD.

The increase in pennation angle observed after 12 weeks was significant in both groups (i.e., RT1: 17.5 ± 9.8%, RT3: 36.3 ± 15.5%) and comparable to previous reports (Abe et al., 2020). The implications of a larger pennation angle are that more contractile material can be accommodated along the length of tendons which, in turn, can generate a larger physiological cross-sectional area (PCSA) and greater muscle strength. Notably, intervention studies that observed smaller (yet significant) increases in pennation angle than those reported in our investigation, were less varied in terms of the “lower body” component of RT (e.g., 10.8% after 12 weeks of unilateral leg extensions only) (Wakahara et al., 2015) or used a substantially shorter intervention (e.g., 7.7% after 35 days of training) (Seynnes et al., 2007). In this respect, it has been shown that muscle hypertrophy does not usually occur in the first 4 weeks of RT, and that remodelling of muscle architecture precedes increases of ACSA (Seynnes et al., 2007). The proposed 4-week delay in muscle adaptations may be even more relevant in deconditioned individuals, since the hypertrophic adaptations of older and/or frail adults are compromised compared to young and healthy individuals (Bickel et al., 2011).

The current study did not reveal any significant changes in fascicle length over time and between groups. This observation is aligned with findings from a review of RT studies that reported enlargements of pennation angles (and PCSA) but limited or no changes in fibre length (Blazevich 2006). The lack of observable effects on fibre length would also seem to indirectly suggest that the larger pennation angle found after the intervention contributed to the greater VL ACSA. While using the pennation angle as a direct proxy measure for PCSA may lead to biased findings, it could be theorised that the PCSA would have grown by at least 28% (i.e., pennation angle increase: RT1 = 17.5%, RT3 = 36.3%, overall = 28.2%) with concomitant increase of maximal strength. This would be compatible with the strength increases highlighted in the study (LPPF = 57%, KEPF = 30%).

Several studies have proposed that neural drive during a maximal contraction increases rapidly within a few weeks of RT, as evidenced through an increase of integrated electromyographic activity (Škarabot et al., 2021). Consequently, the improvements in strength and physical function found in the current study may reflect the initial neural adaptations, which are thought to occur during the first 6 weeks of RT (Blazevich, 2006). Following this initial period, muscle hypertrophy would theoretically have greater impact on measures of strength.

Interestingly, the improvements in LPPF were also reflected in the STS test results, which employed the same movement pattern used in the RT intervention. Notably, participants in both groups exhibited significant improvements in STS60 and STS5 performance greater than established minimal detectable changes (i.e., four repetitions and 2.93 s, respectively) reported in the literature (Segura-Ortí and Martínez-Olmos, 2011; Muñoz-Bermejo et al., 2021). Although the movements performed during the STS60 and STS5 are the same, the STS60 test has a greater endurance component and resulted in greater improvement compared to the STS5, which emphasises speed of execution instead. This finding is in apparent disagreement with findings from a systematic review of exercise in CKD (Heiwe and Jacobson, 2011) that reported no improvements in STS60 and muscle endurance after 6 months of high intensity RT. However, the improvement of STS60 performance observed in our investigation may be explained by the fact that the RT intervention focussed on repeating movements in a steady and controlled manner, without emphasising the speed of execution. In addition, the RT may have benefitted the efficiency of muscular oxygen uptake and allowed participants to use the muscles more fully (Berg et al., 2018). Importantly, the improvements shown in the NSRI walk test may reflect this, since the NSRI has been validated as a proxy of VO_2_ peak in individuals living with CKD (Mercer et al., 1998). The increase in Physical Activity Recall after the 12 weeks also seems to indicate that participants tended to increase activities in daily life which, in turn, may have also marginally improved the physical function tests.

All components of the LUSS (number, frequency, intrusiveness of uraemic sypmtoms and total score) were improved in both RT1 and RT3 after the intervention, which underscores an improvement in health-related quality of life. An observational study of quality of life in people with CKD highlighted differences in LUSS scores based on disease status (e.g., normal kidney function vs. moderate CKD vs. advanced CKD) (Pugh-Clarke et al., 2006). In a more recent study, Brown et al. (2017) reported a median number of five and six uraemic symptoms (assessed through the LUSS) in individuals living with CKD-1/2 and CKD-3, respectively. Notably, the reduction of uraemic symptoms observed at the end of this RT intervention (Table 2) would be compatible with a change from moderate/advanced CKD levels (i.e., CKD-3) to those typically observed in people with CKD with normal kidney function (i.e., CKD-1/2) (Brown et al., 2017). The benefits of exercise compared to no-exercise on uraemic symptomatology had already been documented in a study by Kosmadakis et al. (2012). In this study, authors reported significant reductions in frequency and intrusiveness of symptoms, as well as in total LUSS score, following a walking intervention (30 min/day, five times/week for 6 months) in people with CKD-4. However, in our study, the 12-week RT programme significantly improved all components of the LUSS, including a reduction in the number of symptoms (LUSS1, *p* < 0.05) with fewer training sessions (i.e., only one session per week required) compared to the walking intervention implemented by Kosmadakis et al. (2012). It is also possible that RT may be more efficacious than walking in ameliorating LUSS scores, as performing strengthening exercises may have a more favourable effect on symptoms such as “loss of muscular strength or power” or “restless legs, muscle spasm or stiffness, and pain in joints”. Overall, our results strongly suggest that RT is a viable strategy to reduce, and almost normalise, common uraemic symptoms experienced by people living with CKD-3.

### 4.1 Study Strengths and Limitations

The present study is not without limitations. First of all, we should acknowledge that the heterogeneity in some of the baseline characteristics (e.g., age and weight, Table 1), combined with the relatively small sample size (*n* = 17), may partially account for the lack of statistically significant findings from the intervention. We should also acknowledge that the lack of statistically significant findings in some of the outcome measures (e.g., maximal strength) may be confounded by residual fatigue arising from the 12-week RT intervention and/or by the lack of training monitoring data. Moreover, we did not include information on the aetiology of CKD, disease duration, and medications used by participants. The lack of this information could be construed as a study limitation and may be indicative of randomisation bias. In addition, the absence of a control group consisting of non-exercisers represents a study limitation. Nevertheless, the upper body measurements enabled us to have surrogate control data for the RT programme. Particularly, the lack of favourable change in upper body strength and muscle size indicates that the changes observed in the primary outcome measures is probably attributed to the intervention rather than to any other systemic mediated changes.

On the other hand, all the recorded significant changes following the RT intervention were greater than established error/minimal detectable changes documented in the literature (e.g., NSRI walk (Mercer et al., 1998), STS5 (Muñoz-Bermejo et al., 2021), STS60 (Segura-Ortí and Martínez-Olmos, 2011), muscle and fat ultrasound outcomes (Geneen et al., 2022)), which highlights the significance of our findings. In addition, the study has clinical relevance as it contributed to addressing an existing knowledge gap regarding the identification of optimal RT training frequencies for ameliorating established measures of muscle wasting and function in the CKD-3 population.

Lastly, the study did not examine whether the improvements of strength, muscle size/architecture, physical function, and uraemic symptomatology were maintained following the intervention, which limits our understanding of the long-term effects of the RT programme used in our investigation.

## 5 Conclusion

Findings from the present study suggest that, to obtain greater gains in measures of muscle size and architecture (ACSA and pennation angle), performing RT three times per week is more beneficial than one time per week in people with CKD-3. However, this was not observed for measures of strength, physical function, and perceived uraemic symptomatology as their responses were similar whether training three times or one time per week. Therefore, performing RT just once weekly may represent a viable pre-habilitation strategy for NDD populations, where improved physical function, and health-related quality of life are very common targets. Further research with long-term follow-up, and including a comparison with usual care, would allow us to better understand whether a similar RT programme could delay dialysis initiation and frailty in people with moderately reduced kidney function.

## Data Availability

The raw data supporting the conclusion of this article will be made available by the authors, without undue reservation.

## Abbreviations

ACSA: anatomical cross-sectional area; BMI: body mass index; CKD: chronic kidney disease; CKD-3: stage-3 chronic kidney disease; GFR: glomerular filtration rate; KEPF: knee extension peak isometric force; LPPF: leg press peak isometric force; LUSS: Leicester Uraemic Symptom Score; NDD: non-dialysis dependent; NSRI: North Staffordshire Royal Infirmary; PCSA: physiological cross-sectional area; RM: repetition maximum; RT: resistance training; RT1: resistance training once per week; RT3: resistance training three times per week; STS5: sit-to-stand 5; STS60: sit-to-stand 60; VL: vastus lateralis.
